# Prognostic value of epidermal growth factor receptors in gastric cancer: a survival analysis by Weibull model incorporating long-term survivors

**DOI:** 10.1007/s10120-013-0236-z

**Published:** 2013-02-28

**Authors:** Alexandre Andrade Anjos Jácome, Durval R. Wohnrath, Cristovam Scapulatempo Neto, Estela C. Carneseca, Sérgio V. Serrano, Luciano Souza Viana, João S. Nunes, Edson Z. Martinez, José Sebastião Santos

**Affiliations:** 1 Department of Medical Oncology, Barretos Cancer Hospital, Str. Antenor Duarte Villela, 1331, Barretos, SP 14784-400 Brazil; 2 Department of Gastrointestinal Surgical Oncology, Barretos Cancer Hospital, Str. Antenor Duarte Villela, 1331, Barretos, SP 14784-400 Brazil; 3 Department of Pathology, Barretos Cancer Hospital, Str. Antenor Duarte Villela, 1331, Barretos, SP 14784-400 Brazil; 4 Center for Researcher Support, Barretos Cancer Hospital, Str. Antenor Duarte Villela, 1331, Barretos, SP 14784-400 Brazil; 5 Department of Social Medicine, School of Medicine, University of São Paulo at Ribeirão Preto, Av. Bandeirantes, 3900, 2nd Floor, Ribeirão Preto, SP 14049-900 Brazil; 6 Department of Surgery and Anatomy, School of Medicine, University of São Paulo at Ribeirão Preto, Av. Bandeirantes, 3900, 9th Floor, Ribeirão Preto, SP 14049-900 Brazil

**Keywords:** Stomach neoplasms, Epidermal growth factor receptor, HER2, Survival analysis, Microarray analysis

## Abstract

**Background:**

There is no consensus about the prognostic role of HER2 expression and that of other members of the EGFR family in gastric cancer patients. The aim of this study was to evaluate the prognostic value of the EGFR family in gastric cancer.

**Methods:**

This retrospective study included 201 patients with gastric and esophagogastric junction adenocarcinoma stages 0–IV (AJCC 6th edition) who underwent primary tumor resection. Tissues from primary tumors were analyzed by tissue microarray technology and immunohistochemistry. Correlations between receptor expression and clinicopathological characteristics were performed according to the chi-square test. Survival analysis was calculated according to the Weibull model with a mixture model incorporating long-term survivors. Multivariate analysis of prognostic factors was performed by a regression model incorporating long-term survivors with the Weibull distribution.

**Results:**

Membrane expression of HER1, HER2, and HER4 were 9, 17, and 15 %, respectively. No membrane expression of HER3 was observed. Cytoplasmic expression of HER1, HER3, and HER4 were 45, 62, and 24 %, respectively. HER2 and HER3 expression were correlated (*p* < 0.001) and associated with intestinal-type histology (*p* = 0.001 and *p* < 0.001, respectively) and advanced age (*p* = 0.011 and *p* = 0.008, respectively). According to a regression model adjusted for age, surgical radicality, surgical modality, Laurén histology, adjuvant therapy, TNM stage, and receptor expressions, only TNM stage showed prognostic influence.

**Conclusions:**

According to analysis by a parametric model, the EGFR family did not have prognostic influence in the gastric cancer population studied. The data presented showed a correlation between HER2 and HER3 expression, which might suggest a potential role for HER2–HER3 heterodimerization inhibitors.

## Introduction

The epidermal growth factor receptors HER1 (also denoted EGFR), HER2, HER3, and HER4 are involved in the pathogenesis and progression of solid tumors such as cancer of the breast, lung, bladder, colon, ovary, and stomach [1–3]. All these receptors, except HER3, share the same molecular structure, with an extracellular domain that binds to the ligand, a transmembrane portion, and an intracellular domain with tyrosine kinase activity.

The binding of different ligands to extracellular domains triggers intracellular signaling reactions involved in cell differentiation, proliferation, and survival. The binding of the ligand to the extracellular domain induces HER1 homodimerization and heterodimerization of the remaining receptors, especially HER2 [4, 5].

HER2 overexpression or amplification has a well-established prognostic role in breast cancer and is a predictive factor of the response to drugs that act on the receptor, such as trastuzumab and lapatinib [6, 7]. In gastric cancer, phase II studies have demonstrated the benefits of the use of trastuzumab and lapatinib for locally advanced and metastatic disease with HER2 overexpression or amplification [8, 9]. A phase III study has recently demonstrated a gain in overall survival with the addition of trastuzumab to chemotherapeutic treatment in patients with HER2-positive advanced gastric cancer, supporting the role of this receptor as a predictive factor of the response to anti-HER2 drugs, although its prognostic role is still uncertain [10–14].

In addition to HER2, HER1 and HER3 have also been pointed out as prognostic factors in gastric cancer, although with important caveats regarding the methodological resources for evaluation [10, 12, 15–18]. HER4 has been little studied so far in gastric cancer, but seems to have different effects on survival according to the tumor evaluated [19–21].

The objective of the present study was to contribute to the investigation of the prognostic role of different receptors belonging to the EGFR family in patients with gastric cancer.

## Patients and methods

This was a retrospective study involving 201 patients with stage 0–IV gastric and esophagogastric junction (EGJ) carcinomas with distant metastases (AJCC, 6th edition), who underwent gastrectomy or esophagogastrectomy during the period from 1 January 2006 to 21 December 2008 at the Barretos Cancer Hospital, Barretos, São Paulo, Brazil (Table 1) and for whom surgical specimens were available for protein determination.

**Table 1:** Characteristics of the patients

Characteristics	Number (%)
Total	201 (100)
Gender	
Male	124 (62)
Female	77 (38)
Age	
Median	62
Range	27–88
Tumor location	
Stomach	160 (81)
EGJ	38 (19)
Laurén’s histology	
Intestinal type	124 (63)
Diffuse type	57 (29)
Mixed type	16 (8)
Surgical resection	
R0	150 (75)
R1	16 (8)
R2	33 (17)
Type of lymphadenectomy	
D0	5 (3)
D1	31 (15)
D2	126 (63)
Not related	39 (19)
Lymph nodes	
Median	20
Range	2–69
Tumor depth	
pTis	3 (2)
pT1	18 (9)
pT2	32 (16)
pT3	130 (65)
pT4	18 (9)
Nodal status	
N0	69 (35)
N1	66 (34)
N2	41 (21)
N3	21 (11)
TNM stage	
0	3 (2)
IA	15 (8)
IB	19 (10)
II	38 (19)
IIIA	49 (25)
IIIB	25 (13)
IV M0	19 (10)
IV M1	30 (15)
Adjuvant therapy	
Surgery alone	76 (38)
Chemoradiotherapy	125 (62)

After surgical treatment or adjuvant chemoradiotherapy, the patients were followed up with medical visits, physical examination, laboratory tests, and a chest X-ray at 3-month intervals during the first 2 years, at 4-month intervals during the third year, biannually during the fourth and fifth years, and annually after the fifth year. The patients were submitted to abdominal ultrasonography at 4-month intervals during the first 2 years, at 6-month intervals from the third year on, and annually after the fifth year. Computed tomography, nuclear magnetic resonance, and upper digestive endoscopy were performed based on clinical criteria.

The protein expression of the receptors was related to clinical and pathological characteristics such as age, Laurén histological classification, tumor depth, nodal metastases, TNM stage and overall survival.

Overall survival was defined as the time, in months, that elapsed from the date of surgery to the date of death from any cause. The patients lost to follow-up were censored on the date of last contact with the hospital. The study was approved by the Institutional Review Board of the hospital.

### Tissue microarray (TMA)

Tissue samples were fixed in buffered 4 % formalin, embedded in paraffin, and used for TMA construction as described [22]. A slide with a representative tumor was selected, and an area of the tumor was circled on the slide. Using TMA technology (Beecher Instruments, Silver Spring, MD, USA), the area of interest in the donor block was cored twice with a needle 1.0 mm in diameter and the core transferred to a recipient paraffin block.

### Immunohistochemistry (IHC)

TMA sections were stained with primary antibodies: HER1 [H11 mouse monoclonal (Dako, Carpinteria, CA, USA); dilution, 1:100], HER2 [A0485 rabbit polyclonal (Dako); dilution, 1:1500], HER3 [RB-9211 rabbit polyclonal (*N*terminal; Neomarkers, Fremont, CA, USA); dilution, 1:100], and HER4 [RB-9045 rabbit polyclonal (C-terminal; Neomarkers); dilution, 1:300]. A standard peroxidase-conjugated streptavidin–biotin method was used to detect the staining reaction (LSAB+; Dako). External positive control tissues included samples of placental tissues positive for the antibodies studied. For negative controls, primary antibodies were omitted and phosphate-buffered saline was substituted. Staining was evaluated by light microscopy and interpreted by a pathologist who was blind to the clinical information. Membrane staining was evaluated for HER1, HER2, HER3, and HER4. Staining of HER1, HER3, and HER4 was classified into four categories (0 no staining, 1+ light staining, 2+ moderate staining, 3+ strong staining) according to established criteria [15, 23]. The recommendations of the consensus panel for HER2 in gastric cancer were used for the classification of HER2 [24, 25]. Cytoplasm staining was evaluated for HER1, HER3, and HER4, and also classified into four categories (0 no staining, 1+ light staining, 2+ moderate staining, 3+ strong staining) according to the criteria used in a similar study [15]. The sections classified as 0 and 1+ were considered to be negative and those classified as 2+ and 3+ were considered to be positive, for both membrane and cytoplasm expression.

### Statistical analysis

**Fig. 1:**
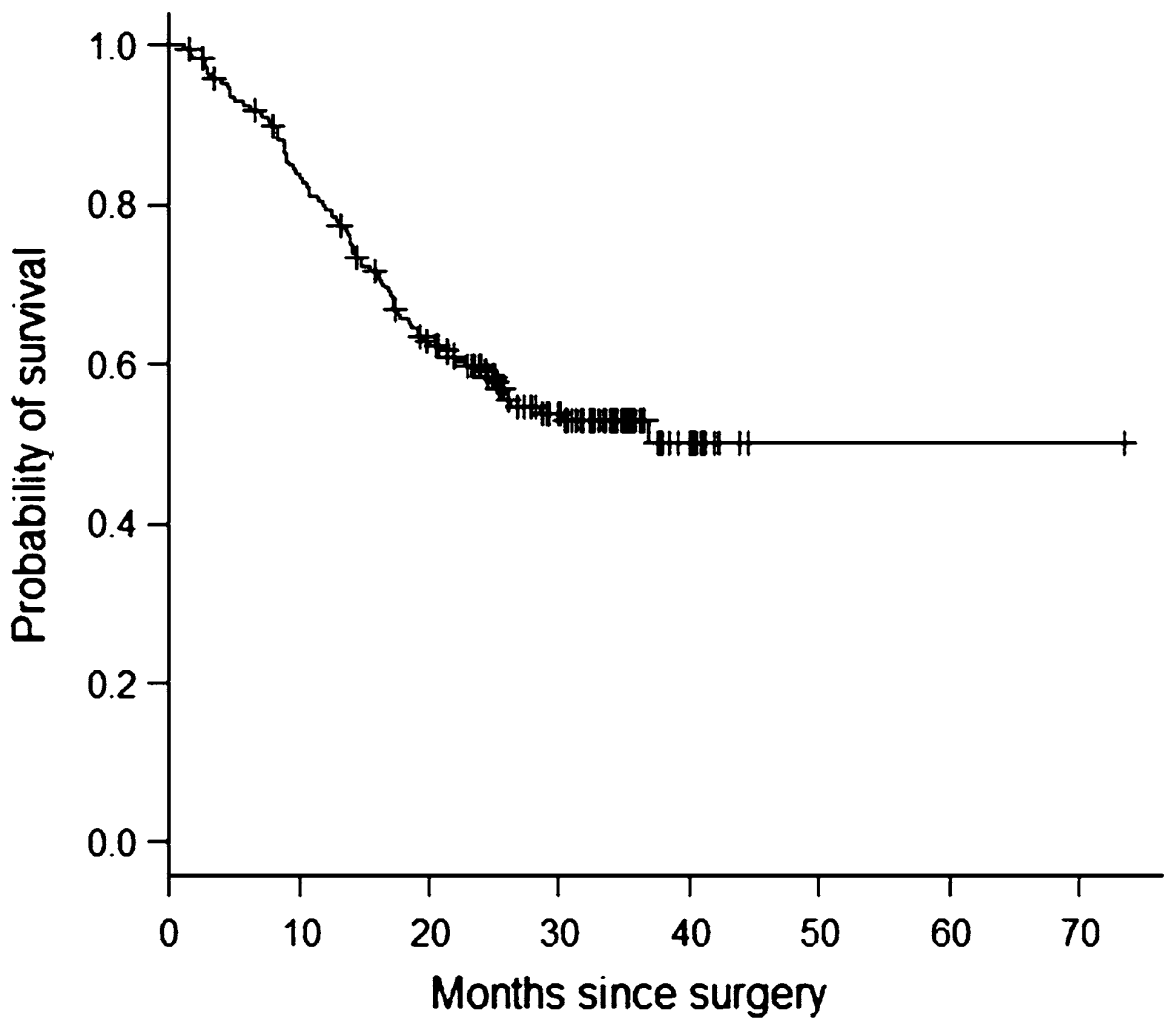
Kaplan–Meier overall survival curve

**Fig. 2:**
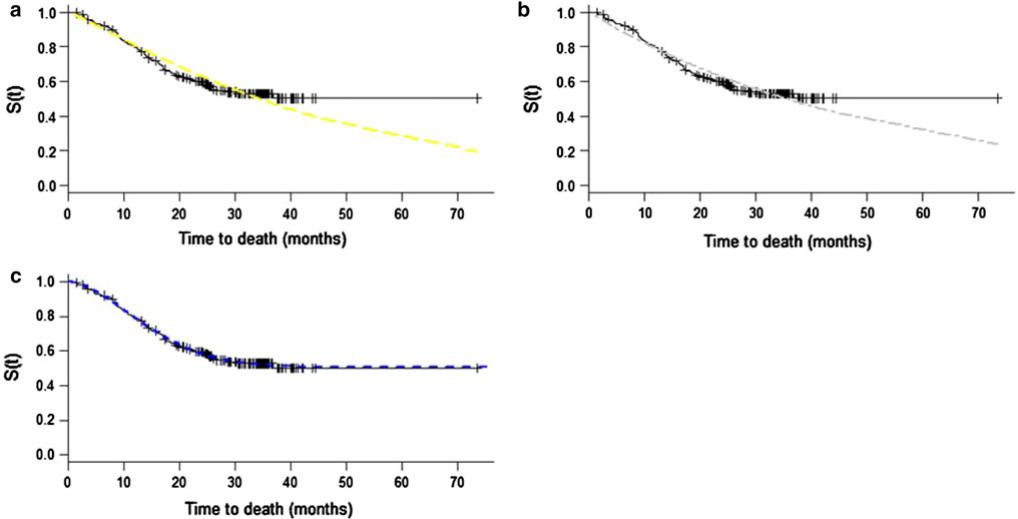
Survival curves estimated by Kaplan–Meier method and parametric models assuming Weibull (**a**), exponential (**b**) and Weibull mixture model incorporating long-term survivors (**c**) distributions

**Fig. 3:**
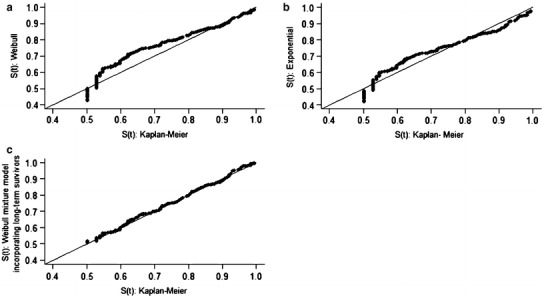
Graphic representation of survival functions estimated by the Kaplan–Meier method versus survival functions estimated by the parametric models assuming Weibull (**a**), exponential (**b**), and Weibull mixture model incorporating long-term survivors (**c**) distributions

Correlations between receptor expression and clinicopathological characteristics were calculated by the chi-square test. Kappa coefficient and McNemar test were utilized to assess concordance among receptor expression. The study sample contained a large number of patients with long-term survival (Fig. 1), a distribution that permitted the use of the Weibull model with a mixture model incorporating long-term survivors for survival analysis [26–28]. Figures 2 and 3 demonstrate the adequacy of this parametric model to the nonparametric Kaplan–Meier model, which permits its use. The regression model incorporating long-term survivors with Weibull distribution was also used for the study of the prognostic variables. Because the proportionality assumption of the Cox model was not held, the Cox regression model was not an appropriate choice for analyzing the present data. *p* values < 0.05 were considered to indicate statistical significance. The analyses were performed using the R software.

## Results

### HER1

A total of 198 samples were available for HER1 analysis: 9 % with positive membrane staining (4 % with a 2+ score and 5 % with a 3+ score) and 45 % with positive cytoplasm staining. There was no correlation between HER1 and age, Laurén histological classification, tumor depth, nodal metastases, or TNM stage.

### HER2

Thirty-four of the 201 samples (17 %) showed positive membrane HER2 staining (11 % with a 2+ score and 6 % with a 3+ score). HER2 positivity was correlated with more advanced age (*p* = 0.011) and with the Laurén’s intestinal type (*p* = 0.001). There was no correlation between HER2 expression and tumor depth, nodal metastases, or TNM stage.

### HER3

Of the 200 samples available for HER3, only 1 showed membrane reactivity. However, 62 % showed cytoplasm positivity, which was related to more advanced age (*p* = 0.008) and to Laurén’s intestinal type (*p* < 0.001). There was no correlation between cytoplasmic HER3 expression and tumor depth, nodal metastases, or TNM stage. The correlation among membrane HER3 expression, clinicopathological characteristics, and overall survival was not evaluated because only one patient showed membrane positivity.

### HER4

**Fig. 4:**
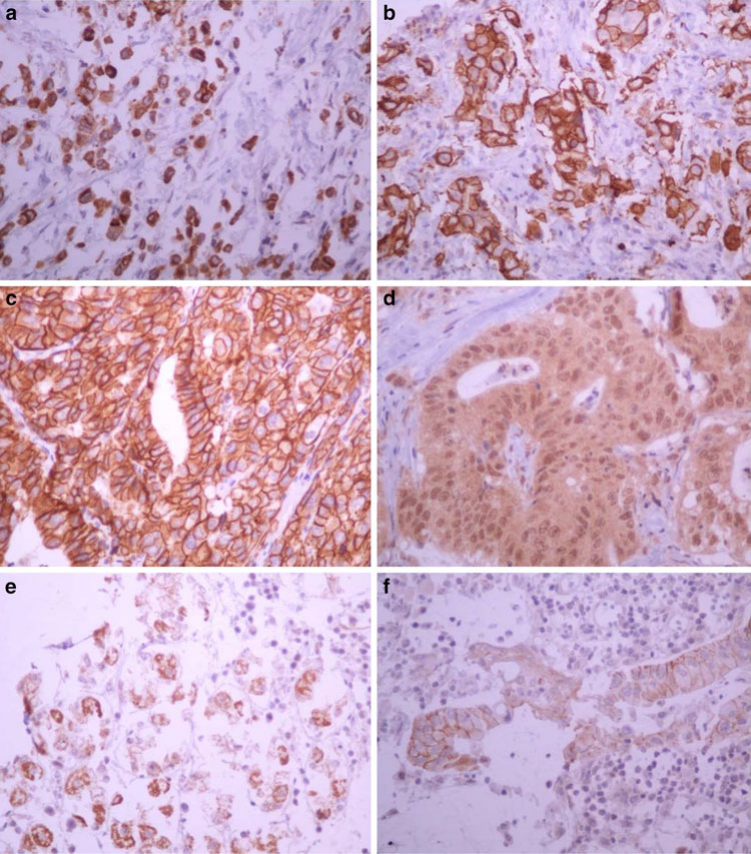
Examples of positive immunohistochemistry for HER1 staining in the cytoplasm (**a**), for HER1 in the membrane (**b**), for HER2 in the membrane (**c**), for HER3 in the cytoplasm (**d**), for HER4 in the cytoplasm (**e**), and for HER4 in the membrane (**f**). Magnification: **a–f** ×400

Twenty-nine of the 199 samples available (15 %) showed positive membrane HER4 staining, with a 2+ score in 10 % and a 3+ score in 5 %; 24 % of these showed positive cytoplasm staining. There was no correlation between HER4 expression and age, Laurén histological classification, tumor depth, nodal metastases, or TNM stage. Examples of positive immunohistochemistry for HER1, HER2, HER3, and HER4 are shown in Fig. 4.

### Receptors and overall survival

There was a concordance among expression of the EGFR family receptors, with the exception of membrane HER1 and HER4, and membrane HER2 and HER4 (Table 2).

**Table 2:** Concordance between membranous and cytoplasmic expression of HER receptors

	HER1 (cytoplasm)				HER2 (membrane)				HER3 (cytoplasm)				HER4 (membrane)				HER4 (cytoplasm)			
	Pos	Neg	*k* ^a^	*p* ^b^	Pos	Neg	*k* ^a^	*p* ^b^	Pos	Neg	*k* ^a^	*p* ^b^	Pos	Neg	*k* ^a^	*p* ^b^	Pos	Neg	*k* ^a^	*p* ^b^
HER1 (membrane)																				
Pos	13	4	0.115	<0.001	5	12	0.092	0.008	13	4	0.042	<0.001	4	13	0.279	0.052	5	12	0.028	<0.001
Neg	77	104			29	152			109	71			25	156			44	137		
HER1 (cytoplasm)																				
Pos					17	73	0.033	<0.001	58	31	0.057	<0.001	10	80	0.0	<0.001	27	63	0.100	<0.001
Neg					17	91			64	44			19	89			22	86		
HER2 (membrane)																				
Pos									23	11	0.033	<0.001	7	27	0.077	0.475	14	20	0.170	0.043
Neg									101	65			22	143			35	130		
HER3 (cytoplasm)																				
Pos													24	99	0.103	<0.001	35	88	0.082	<0.001
Neg													5	70			14	61		
HER4 (membrane)																				
Pos																	13	16	0.184	0.006
Neg																	36	134		

^a^Kappa coefficient

^b^McNemar test

Receptor expression did not differ according to disease stage (Table 3).

**Table 3:** Positivity rates of HER receptors according to TNM stage

TNM stage	HER 1 (membrane)	HER1 (cytoplasm)	HER2 (membrane)	HER3 (cytoplasm)	HER4 (membrane)	HER4 (cytoplasm)
0/I/II	6/72 (8 %)	31/72 (43 %)	14/75 (19 %)	43/75 (57 %)	12/73 (16 %)	20/73 (27 %)
III/IV	9/93 (10 %)	44/93 (47 %)	15/93 (16 %)	56/92 (61 %)	11/93 (12 %)	24/93 (26 %)
IVM1^a^	1/30 (3 %)	14/30 (47 %)	5/30 (17 %)	23/30 (77 %)	4/30 (13 %)	5/30 (17 %)
*p*	0.545	0.258	0.907	0.176	0.692	0.321

^a^Metastatic disease

Patients who died during the postoperative period (*n* = 12) were excluded from survival analysis, with 189 patients remaining in the study. Eighty-three patients (43.9 %) died and 106 (56.1 %) were censored in a median follow-up time of 30.26 months.

**Fig. 5:**
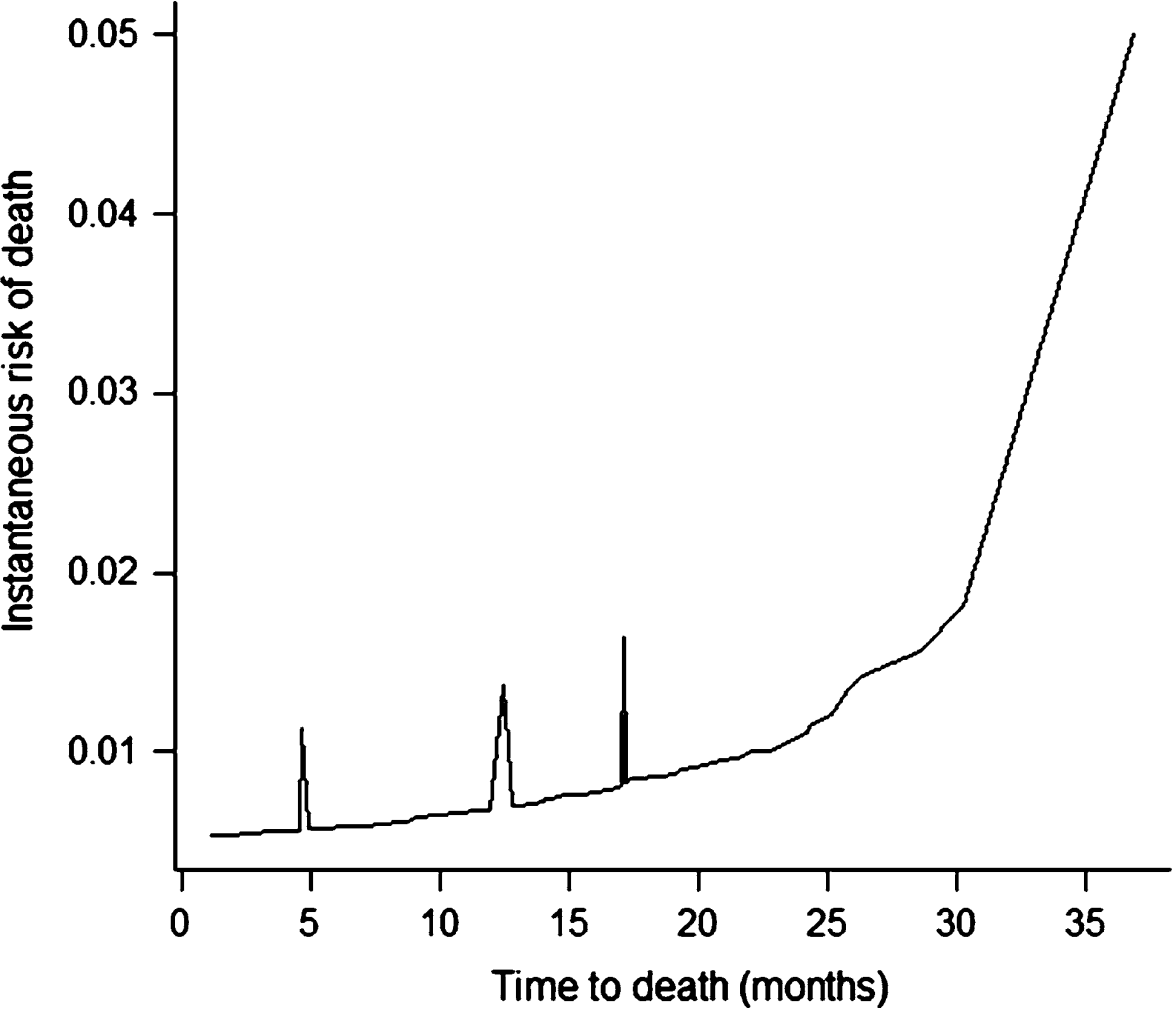
Instantaneous risk of death

The exploration of the Weibull model incorporating long-term survivors allowed the estimation of cure fraction, estimated at 51 % [95 % confidence interval (CI), 0.42–0.59]. It was also possible to evaluate the instantaneous risk, which demonstrated an increasing risk of death with time, but was more impressive at 4, 12, and 17 months after surgical treatment in the sample studied (Fig. 5).

**Fig. 6:**
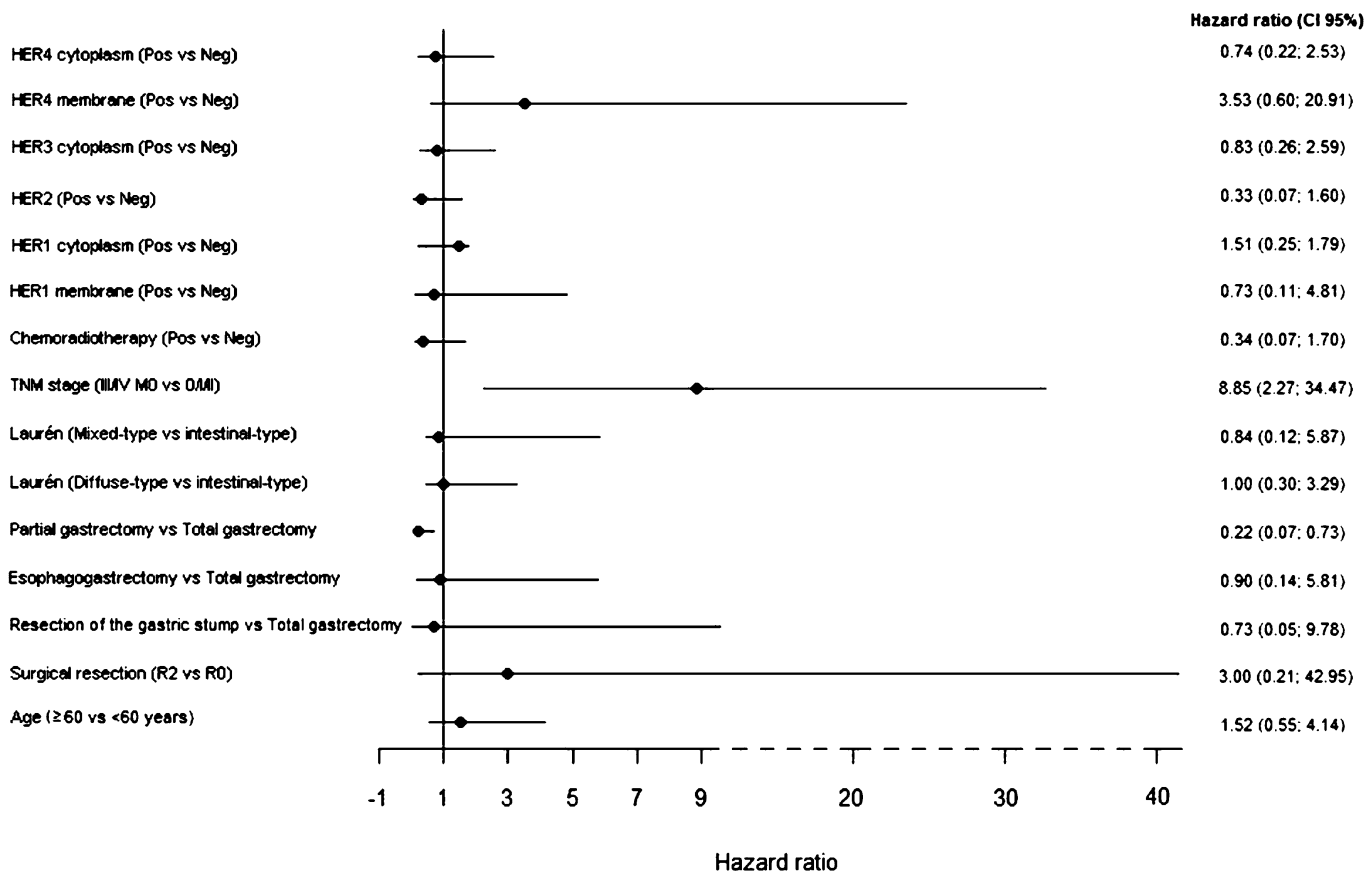
Estimation of parameters of the regression model incorporating long-term survivors with Weibull distribution and related covariates

The regression model incorporating long-term survivors with Weibull distribution adjusted for age, surgical radicality, type of surgery, Laurén histological classification, adjuvant treatment, TNM stage, and cell receptors revealed that TNM stage was the only variable with a prognostic influence (Fig. 6).

## Discussion

The samples studied represent a heterogeneous population consisting of patients with localized and metastatic gastric adenocarcinoma, with different histological subtypes and subjected to adjuvant chemoradiotherapy or surgery alone. However, the methodology used in the present study did not demonstrate a prognostic value of receptors of the EGFR family in patients with gastric cancer who underwent gastrectomy, as also observed in two major studies that evaluated the prognostic value of the EGFR family in gastric cancer [15, 17].

Positivity for the expression of the EGFR family receptors depends on the sample studied and the methodology used. Factors such as prevalence of Laurén histological type, population age, quality of the samples used, antibodies employed, criteria adopted, and different methodologies among studies are probably responsible for the variability detected in the literature.

A study conducted on a Japanese population that also had heterogeneous characteristics regarding disease stage and further treatment received by the patients, adopting the same criteria for the classification of receptors of the EGFR family as used in the present study but without immunohistochemical evaluation by TMA, suggested that HER3 may also have a prognostic influence on gastric cancer [14].

Evaluation in a Western population, also with localized and metastatic disease but without the use of adjuvant treatment, detected a prognostic influence of HER2 and HER3 expression by univariate analysis; however, this was not reproduced by multivariate analysis [10, 17]. This study [17], similar to the present one, used TMA for the reading of the immunohistochemical analysis, but employed different criteria for the interpretation of HER1, HER3, and HER4 expression, in addition to also employing fluorescence in situ hybridization (FISH).

There still is no consensus about the criteria to be adopted for the reading of these three receptors in gastric cancer that resembles those available for the interpretation of HER2 expression [24, 25]. The rates of expression of the receptors observed in the present study were similar to those of the study conducted on a Western population, whereas the study on the Japanese population demonstrated much higher levels of HER1 expression in the membrane and of HER4 in the cytoplasm (Table 4). So far, there are no data indicating that race and geographic location may be factors responsible for this variability.

**Table 4:** Comparison of rates of HER receptor expression in gastric cancer

Receptors	Hayashi et al. [15]	Begnami et al. [17]	Jácome et al. [53]
HER 1 (membrane) (%)	30	2	9
HER1 (cytoplasm) (%)	NR	NR	45
HER2 (%)	18 (IHC)	12 (IHC) 8 (FISH)	17 (IHC)
HER3 (membrane) (%)	13	<1	<1
HER3 (cytoplasm) (%)	58	64	62
HER4 (membrane) (%)	22	18	15
HER4 (cytoplasm) (%)	84	23	24

*IHC* immunochemistry, *FISH* fluorescence in situ hybridization, *NR* not related

The expression of HER1 in gastric cancer ranges from 2 to 44 % [29–32], and the 9 % positivity observed in the present study is within recorded limits. The prognostic value of its expression is controversial, with some data even suggesting that HER1 overexpression may predict a higher risk of disease recurrence after adjuvant treatment with platinum and fluoropyrimidine [32, 33].

The rate of HER2 expression in gastric cancer ranges from 8 to 34 %, with a mean of 17.6 % [24]. Recent systematic review without meta-analysis involving more than 11,000 patients showed 18 % of HER2 overexpression and suggests a poorer overall survival for these patients [34]. The 17 % positivity and the frequent association between HER2 expression and Laurén’s intestinal type were also observed in the present study.

In the present study there was also an association between HER2 overexpression and advanced age, in agreement with the findings of a recent study that detected a rate of HER2 overexpression of only 3 % and a rate of HER2 amplification of 5 % in patients younger than 45 years [35]. These data support the hypothesis that gastric cancer of early onset has a different profile of molecular expression than disease of late onset [36, 37].

In the present sample, HER3 as well as HER2 overexpression was associated with Laurén’s intestinal type and advanced age. Studies on HER3 expression in gastric cancer are still scarce, but the association with Lauren’s histological type is controversial, with a relationship having been detected with both the intestinal type [17] and the diffuse type [16]. The association with advanced age had not been reported previously [15–17]. In the present study, the association between HER2 and HER3 expression, and the finding of similar clinicopathological associations between the expression of these two receptors, contributes to the hypothesis that, among the heterodimers of the EGFR family, these two receptors are those expressed at high frequency [17]. This finding is of relevant importance in the signaling of the phosphatidyl inositol-3 kinase pathway [38, 39], a fact that makes HER3 a potential target for the treatment of gastric cancer.

The absence of tyrosine kinase activity of HER3 initially led to the idea that this is a receptor of minor importance in cell proliferation and differentiation, but increasing evidence has demonstrated its role as an important regulator of HER2 activity [40]. The benefit demonstrated by the addition of pertuzumab—a drug that inhibits HER2-HER3 heterodimerization—to trastuzumab in the treatment of HER2-positive breast cancer supports the importance of this heterodimer in the proliferation of tumor cells with HER2 overexpression or amplification [41] and suggests this new monoclonal antibody is a potentially effective agent for the treatment of gastric cancer [42].

HER4 did not show a prognostic value in the present study or in similar investigations [15, 17]. Some data regarding breast cancer have suggested that the expression of receptors of the EGFR family should not be analyzed and interpreted separately as distinct units. The prognostic value of these receptors is probably determined by their interrelationship [21, 43].

Coupling the distribution of survival data to a predefined model permits their analysis by parametric models, which are known to allow a more detailed and reliable interpretation of the data [44]. During long-term follow-up and in the presence of a significant group of long-term survivors, the nonparametric models lose their power of analysis and should be preferentially avoided [45]. A frequent occurrence in survival analysis is the detection of individuals who, after a long follow-up period, do not present the occurrence of the event of interest. In the present study, we chose to analyze the data using a parametric model in view of the adaptation of the distribution of survival data to the Weibull model with a mixture model incorporating long-term survivors, which permitted the incorporation of individuals with a low probability of death as the event of interest.

Although the Cox model is the method most frequently used in analyses involving time until a given event, the assumption of risk proportionality among the categories of a given covariable is not always satisfied. Adjustment to the Weibull model demonstrated the flexibility of the parametric models regarding the easy incorporation of the effects of covariables in their parameters, in addition to the ability to provide more information about the nature of the distribution of survival time and of the behavior of the risk function along time, data that nonparametric or semiparametric models do not provide [46]. According to the reporting recommendations for tumor marker prognostic studies, the biological markers were included in the model of multivariate analysis, with a parametric regression model being used [10, 47].

The heterogeneity of HER2 expression in gastric cancer leads to questioning the value of TMA as a method for the assessment of HER2 status in this neoplasia. The evaluation of expression and amplification using samples that contain a greater portion of tumor tissue may perhaps be more representative for the test and may reduce the probability of false-negative results. However, this method has been extensively used for the reliable detection of biomarkers, including those with heterogeneous distribution in tumor tissue [48]. In addition, this method simulates the gastric biopsies performed by upper digestive endoscopy, which have well-defined criteria for the evaluation of HER2 status and have been used in various studies [17, 49, 50]. On the other hand, there is a need for additional studies that will validate TMA as an appropriate method for the evaluation of HER2 status.

Despite the high level of concordance between IHC and in situ hybridization methods to evaluate HER2 expression in gastric cancer [51], the current recommendations suggest that samples of patients with IHC 2+ should be referred to in situ hybridization techniques [52]. In the present study, the silver-enhanced in situ hybridization (SISH) method was used, but, perhaps because of the long storage time of the formalin-fixed paraffin-embedded blocks, the reading was not of sufficiently high quality to be reported, which constitutes a limitation of the present study.

The absence of prognostic value of HER2 in gastric cancer demonstrated in some studies does not exclude the predictive value of this receptor regarding anti-HER2 therapies, as demonstrated by the ToGA study [11]. In breast cancer, this receptor has been demonstrated to be a prognostic and predictive marker of benefit regarding anti-HER2 therapies, but, since the introduction of trastuzumab, HER2 expression is no longer a prognostic marker [6]. Large prospective trials with a validated methodology are needed to determine the real prognostic value of HER2 overexpression.
